# A Multi-year Review of Major Off-Road Vehicle Injuries and Deaths in New Brunswick

**DOI:** 10.7759/cureus.80503

**Published:** 2025-03-13

**Authors:** Liam Priest, Andrew Flewelling, Paul Atkinson, Allison Chisholm, Susan Benjamin, Tushar Pishe, Kavish Chandra

**Affiliations:** 1 Emergency Medicine, Dalhousie Medicine New Brunswick, Saint John, CAN; 2 Research Services, Horizon Health Network, Saint John, CAN; 3 Trauma, New Brunswick Trauma Program, Saint John, CAN

**Keywords:** injury, injury patterns, new brunswick, off-road vehicle, public health, trauma

## Abstract

Introduction: Off-road vehicles are widely used in Canada, especially in rural areas. Despite their popularity, there is limited data on off-road vehicle-related injuries, particularly in New Brunswick (NB). The aim of this study was to describe the epidemiology of off-road vehicle incidents in NB, focusing on frequency, severity, and factors associated with morbidity over an eight-year period (2014-2021).

Methods: We conducted a retrospective observational study using data from the New Brunswick Trauma Registry, which includes all patients admitted to level 1, 2, and 3 trauma centers with severe injuries. We analyzed data on demographics, incident characteristics, substance use, helmet use, and outcomes. Regression analyses assessed factors predicting intensive care unit admission and hospital length of stay.

Results: Between December 2014 and December 2021, 681 patients recorded in the trauma registry were involved in off-road vehicle incidents. The median age of these patients was 38.0 years, with 533 patients (78.3%) identified as male and 77 (11.3%) under 18 years old. All-terrain vehicles were involved in 456 (67%) of the incidents, while 225 (33%) involved snowmobiles. Among those tested, 176 (54.2%) had used alcohol, and 390 (57.3%) had used a helmet. The most frequent types of incidents included off-road travel incidents (43.3%) and rollovers (23.5%). The median Injury Severity Score was 13, and the median hospital length of stay was four days. Regression analysis showed that older age and intoxication were significant predictors of longer hospital stays and increased likelihood of admission to the intensive care unit.

Conclusion: This study describes factors associated with off-road vehicle incidents in New Brunswick. Off-road vehicle trauma in New Brunswick presents a considerable public health challenge, with the male gender, older age, and substance use associated with increased morbidity. Effective injury prevention requires a multifaceted approach, including enhanced education, stricter regulations, and improved enforcement to mitigate the risks associated with off-road vehicle (ORV) use.

## Introduction

Off-road vehicle use is widespread across Canada, particularly in rural areas, where nearly half of injuries occur [1]. The popularity of off-road vehicles has increased markedly in recent years, with a substantial rise in sales during the COVID-19 pandemic [2]. In New Brunswick, a large rural population has ready access to recreational off-road vehicle activities and an extensive network of all-terrain trails.

Despite the high utilization of off-road vehicles in Canada, data on the burden of injuries associated with these vehicles is limited. The largest Canadian study to date focused on youth injuries, finding that 15 percent of injured individuals were under the age of 16 [1]. Another study focused on youth and adult all-terrain vehicle incidents in Newfoundland but did not include snowmobile-related incidents [3]. More recently, an analysis of Canada’s impaired driving registry showed that drivers involved in off-road vehicle incidents frequently had positive blood alcohol levels or were positive for cannabinoids, recreational drugs, or impairing medications [4].

These studies collectively indicate a significant burden of injuries from off-road vehicle use among young people and men and an increasing involvement of substance use in incidents. This study aimed to describe the epidemiology of off-road vehicle incidents in New Brunswick, providing insight into the patterns and factors associated with these injuries.

The results of this article were previously presented at the Canadian Association of Emergency Physicians annual meeting in Toronto, Ontario, Canada, as a poster presentation on May 29, 2023.

## Materials and methods

Study design, setting, and population

We conducted a retrospective observational study of all patients injured in off-road vehicle incidents recorded in the New Brunswick Trauma Registry from December 2014 to December 2021. The study was a provincial trauma registry review. The Trauma New Brunswick office is located at the Saint John Regional Hospital in Saint John, New Brunswick, Canada. The New Brunswick Trauma Registry collects data on all patients who present or are transferred to level 1, 2, and 3 facilities and are admitted within the province of New Brunswick, Canada. The project adhered to the Strengthening the Reporting of Observational Studies in Epidemiology (STROBE) guidelines [5] and received approval from Horizon Health Network’s Human Research Protection Program and REB (File # 101561) with a waiver of consent granted according to Tri-Council Policy Statement 2 (TCPS2) article 5.5A [6].

Data collection

For the purposes of this study, off-road vehicles included all-terrain vehicles, utility-terrain vehicles, and snowmobiles. The study excluded all motorcycle incidents (road or off-road). We obtained our data on incident-related variables and outcomes of interest from the Trauma New Brunswick provincial program through their trauma registry for the period of December 2014 to December 2021.

Outcome measures

Incident-related variables of interest included patient sex, age, intoxicant use (including substance and level of impairment if known), utilization of safety equipment, incident date, time, vehicle type, and mechanism. Outcomes of interest included Injury Severity Score (ISS), Glasgow Coma Scale (GCS), patient disposition (e.g., discharge, admission, admission to ICU, deceased), and mortality.

Data analysis

Continuous variables were characterized using the median and interquartile range, while categorical variables were described using frequencies and percentages. No missing data were imputed. Simultaneous linear and logistic regressions were employed to identify patient and incident factors predicting hospital length of stay and ICU admission, respectively. Due to issues with normality, heteroscedasticity, and linearity, hospital length of stay was log-transformed, with the log-transformed variable used within the linear regression. Independent variables included age, sex, day, month, year, vehicle type, helmet use, and alcohol intoxication. Results are reported as percent change or odds ratios with 95% confidence intervals (CI) for linear and logistic regressions, respectively. Multicollinearity of independent variables was assessed using variance inflation factor (VIF), with a VIF greater than 5 indicating multicollinearity. No independent variables were found to be multicollinear. An α = 0.05 was used for all analyses. Statistical analysis was conducted using the IBM SPSS Statistics for Windows, Version 20 (Released 2020; IBM Corp., Armonk, New York, United States) and R software version 4.2.2 (car package version 3.1-2, R Foundation for Statistical Computing, Vienna, Austria).

## Results

Patient factors

Between December 2014 and December 2021, 681 patients were involved in an off-road vehicle-related incident. The median age of patients was 38.0 years, with 77 (11.3%) patients being under 18 years old and 533 (78.3%) being male (Table 1). Of those who underwent blood alcohol testing, 176 (54.2%) patients had a positive blood alcohol level (BAC), and 60% had levels exceeding 0.05% (threshold of impairment in New Brunswick). Two-thirds (456, 67.0%) of the incidents involved all-terrain vehicles, and 225 (33.0%) involved snowmobiles (Table 1).

**Table 1:** Off-road vehicle trauma patient demographics and outcomes for the years 2014-2021 (N = 681) Data presented as n (%) unless otherwise stated. IQR: interquartile range; BAC: blood alcohol content; ATV: all-terrain vehicle; GCS: Glasgow Coma Scale; ISS: Injury Severity Score

Factor	Outcome
Sex, n (%) male	533 (78.3)
Sex, n (%) female	148 (21.7)
Age, median (IQR)	38.0 (25.0 - 53.0)
n (%) > 19 years	604 (88.7)
Positive BAC, n (%) of those tested (N = 325)	176 (54.2)
Vehicle type	
ATV	456 (67.0)
Snowmobile	225 (33.0)
Position of injured patient, n (%) of drivers	566 (83.1)
Crash type	
Off-road	295 (43.3)
Roll-over	160 (23.5)
Struck	115 (16.9)
All others	51 (7.5)
Unknown/missing/not applicable	60 (8.8)
Use of helmet, n (%)	390 (57.3)
Pre-hospital GCS, median (IQR), n = 418	15.0 (15.0 - 15.0)
GCS at arrival, median (IQR), n = 395	15.0 (15.0 - 15.0)
ISS, median (IQR), n = 274	13.0 (4.0 - 19.3)
Crash timing, n (%) of weekend crashes	386 (56.7)
Season of accident	
Fall	104 (15.3)
Winter	235 (34.5)
Spring	151 (22.2)
Summer	191 (28.0)
Year	
2014	2 (0.3)
2015	114 (16.7)
2016	109 (16.0)
2017	114 (16.7)
2018	86 (12.6)
2019	106 (15.6)
2020	119 (17.5)
2021	31 (4.6)
ED discharge disposition	
Admitted to the ICU	129 (18.9)
Admitted to the ward	389 (57.1)
Sent to the operating room/interventional radiology	92 (13.5)
Direct (from another facility bypassing ED)	67 (9.8)
Died	4 (0.6)
Hospital length of stay, median (IQR)	4.0 (2.0 - 7.0)
Hospital discharge status, n (%) of those that died	16 (2.3)
Discharge destination	
Home	578 (85.0)
Rehabilitation facility (specialized and general)	24 (3.6)
Another acute care facility	58 (8.5)
Died	16 (2.2)
Other	5 (0.7)

Temporal factors

Most incidents occurred on weekends (386, 56.7%), with 252 cases (37.0%) presenting on Saturdays and 134 (19.7%) on Sundays. Winter saw the highest incidence of trauma (235 incidents, 34.5%, Table 1), with February having the peak frequency at 88 incidents (12.9%), followed by March at 75 incidents (11.0%) and January at 72 (10.6%). The majority of trauma patients arrived at the Saint John Regional Hospital (SJRH), the Moncton Hospital (TMH), and the Dr. Everett Chalmer's Hospital (DECH) (Table 2).

**Table 2:** Facility of arrival for trauma patients SJRH: Saint John Regional Hospital; DECH: Dr. Everett Chalmer’s Hospital; HRC: Chaleur Regional Hospital; CRH: Campbellton Regional Hospital; TMH: The Moncton Hospital; ERH: Edmundston Regional Hospital; MRH: Miramichi Regional Hospital; GDH: Dr Georges-L Dumont Hospital; GFG: Grand Falls General

Facility of arrival	N	%
SJRH (level 1)	141	20.7
DECH (level 3)	120	17.6
HRC	66	9.7
CRH	52	7.6
TMH (level 2)	157	23.1
ERH	79	11.6
MRH	41	6.0
GDH	<34	<5
GFG	<34	<5
Total	681	100.0

Outcome variable 

The median Injury Severity Score (ISS) was 13.0 (Table 1). Most patients were admitted to the ward (n = 389, 57.1%), followed by the ICU (n = 129, 18.9%) and operating room/interventional radiology (n = 92, 13.5%). Of the 681 patients, 665 (97.7%) were discharged alive, while 16 (2.3%) did not survive. The most common injuries were rib fractures, clavicular fractures, and lower leg fractures (Table 3). 

**Table 3:** Most frequent injuries from off-road vehicle incidents

Injuries	%
Multiple fracture of unspecified number of ribs, closed	6.9
Fracture of unspecified part of clavicle, closed	3.9
Fracture of lower leg, part unspecified, closed	3.9
Fracture of lumbar vertebra, unspecified level, closed	2.9
Fracture of other and unspecified parts of lumbar spine and pelvis, closed	2.7
Fracture of other and unspecified parts of wrist and hand, closed	2.6
Fracture of femur, part unspecified, closed	2.6
Multiple fractures of unspecified number of ribs, closed	6.9
Fracture of unspecified part of clavicle, closed	3.9
Fracture of lower leg, part unspecified, closed	3.9

Predictive factors

Logistic and linear regression were used to identify associations between patient demographics and accident-related factors and an ICU admission from the emergency department and hospital length of stay, respectively. Using logistic regression, it was found that the odds of ICU admission increased with increasing age (OR: 1.02 (1.00-1.03), p = 0.01) and alcohol intoxication (OR: 3.64 (2.29 - 5.81), p < 0.001) (Table 4). Using linear regression, we identified increasing age (0.5% increase (0.2% - 0.7%), p < 0.001) and alcohol intoxication (14.8% increase (5.5% - 25.0%), p = 0.001) were associated with longer hospital LOS (Table 5). Incidents that occurred in the fall, in relation to those in the summer (11.7% decrease (-21.5% - -0.6%). p = 0.039) and the use of helmets (9.2% decrease (-17.5% - -0.3%), p = 0.044) were associated with shorter hospital LOS (Table 5). 

**Table 4:** Prediction of ICU admission from emergency department following an off-road vehicle incident Logistic regression (outcome variable: ICU admission from ED). Model: χ2 (10, N = 500) = 42.18, p = <0.001; Nagelkerke R2: 0.13. CI: confidence interval; ATV: all-terrain vehicle. Bold denotes significant association with the outcome.

Variable	Odds ratio (95% CI)	p-value
Age	1.02 (1.00 - 1.03)	0.010
Sex, female (reference = male)	0.78 (0.44 - 1.41)	0.415
Day, weekend (reference = weekday)	0.85 (0.54 - 1.35)	0.500
Season (reference = summer)		
Fall	1.04 (0.51 - 2.10)	0.920
Winter	1.19 (0.47 - 2.97)	0.718
Spring	1.03 (0.53 - 2.01)	0.931
Year	0.99 (0.88 - 1.12)	0.886
Vehicle type, snowmobile (reference = ATV)	1.32 (0.59 - 2.97)	0.505
Helmet, yes (reference = no)	0.68 (0.39 - 1.17)	0.159
Intoxication, yes (reference = no)	3.64 (2.29 - 5.80)	<0.001

**Table 5:** Prediction of hospital length of stay (log transformation) following an off-road vehicle incident Linear regression (outcome variable: hospital LOS). Model: R2: 0.08, F(10, 489) = 4.21, p = < 0.001; CI: confidence interval; ref: reference category; ATV: all-terrain vehicle. Bold denotes significant association with the outcome.

Variable	Effect size (95% CI)	% Change (95% CI)	p-value
Age	1.01 (1.00 - 1.01)	0.5 (0.2 - 0.7)	< 0.001
Sex, female (ref = male)	0.98 (0.90 - 1.08)	-1.7 (-10.3 - 7.9)	0.724
Day, weekend (ref = weekday)	0.95 (0.88 - 1.03)	-4.6 (-11.7 - 3.1)	0.237
Season (ref = summer)			
Fall	0.88 (0.79 - 0.99)	-11.7 (-21.5 - -0.6)	0.039
Winter	0.89 (0.77 - 1.04)	-10.6 (-23.4 - 4.4)	0.157
Spring	0.93 (0.84 - 1.04)	-6.9 (-16.4 - 3.8)	0.196
Year	0.99 (0.97 - 1.01)	-1.0 (-3.0 - 1.1)	0.347
Vehicle, snowmobile (ref = ATV)	1.13 (0.98 - 1.30)	13.0 (-1.7 - 29.8)	0.084
Helmet, yes (ref = no)	0.91 (0.83 - 1.00)	-9.2 (-17.5 - -0.3)	0.044
Intoxication, yes (ref = no)	1.15 (1.06 - 1.25)	14.8 (5.5 - 25.0)	0.001

## Discussion

This study is the first to provide a comprehensive analysis of the epidemiology and outcomes of off-road vehicle incidents in New Brunswick, shedding light on injury patterns, demographic trends, and significant risk factors. The median age of injured individuals was 38 years, and a considerable majority were male. There is a strong association between older age, alcohol intoxication, and the increased likelihood of intensive care unit admissions following an off-road vehicle incident. Older age, intoxication, and lack of helmet use were significant predictors of prolonged hospital stays.

Interpretation

The data points to a high-risk profile among off-road vehicle users, particularly men and older adults. Males accounted for more than three-quarters of those injured, a pattern consistent with findings from studies in other provinces [3,7]. This pattern may be related to higher participation rates in off-road vehicle activities among men and potentially greater involvement in higher-risk driving behaviors. The significant association between alcohol use and severe trauma outcomes further supports the need for targeted public health initiatives. In over half of the cases where testing was conducted, patients were found to be intoxicated, underscoring the dangers of operating off-road vehicles under the influence of alcohol or other substances.

Seasonal variations in injury rates also emerged, with the highest number of incidents occurring during winter, particularly in February. This seasonal pattern suggests a correlation with increased snowmobile use during colder months, which presents specific hazards such as icy surfaces, reduced visibility, and obstacles hidden beneath snow cover. While snowmobiles are linked to these seasonal risks, most injuries involve ATVs.

Comparison with previous studies

Our findings align with studies from other Canadian regions, such as Nova Scotia, which have highlighted similar risk factors in off-road vehicle trauma cases [7]. However, a distinct feature of our data is the relatively older median age of trauma patients in New Brunswick, which may be partly due to provincial demographics or regulatory requirements that restrict younger drivers’ access to off-road vehicles or mandate adult supervision [8]. The age factor is significant, as older adults may experience greater vulnerability to severe outcomes due to underlying health conditions and slower physical recovery [9].

Although helmets are well-documented for reducing the risk and severity of head injuries, a large portion of off-road vehicle users in New Brunswick continue to ride without helmets, mirroring trends observed in other provinces [10,11]. 

Strengths and limitations

This study's key strength is its use of provincial-level trauma data from the New Brunswick Trauma Registry, detailing severe injury cases in level 1-3 trauma centers. However, it has limitations. By including admitted patients with moderate to severe injuries, it excludes minor injuries treated in outpatient facilities, possibly underestimating the burden. Fatalities at incident scenes are not captured. In addition, pediatric patients who are transferred to a pediatric trauma center (outside of the province) are not captured, which may also underestimate off-road vehicle-related deaths and pediatric-specific severe injuries.

Clinical and public health implications

These findings hold important implications for clinical practice and public health initiatives both within New Brunswick and across Canada. On a public health level, the study supports the implementation of targeted interventions for high-risk groups, notably men and older adults who use off-road vehicles, as well as seasonal risks. Given the strong protective benefits of helmets, their promotion and increased enforcement could be among the most impactful safety measures for reducing severe injuries in off-road vehicle incidents.

Research implications

Future research could examine the impact of safety policies, including helmet and substance use regulations, on reducing off-road vehicle-related injuries across provinces. 

## Conclusions

This multi-year review of off-road vehicle injuries in New Brunswick reveals critical risk factors driving morbidity, including male gender, older age, and intoxication. The findings underscore the urgent need for a targeted, multi-faceted approach to injury prevention. Emphasizing education, stricter regulations, and enhanced enforcement may help alleviate the public health burden posed by off-road vehicle trauma.
